# Trends in nontuberculous mycobacteria infection in children and young people with cystic fibrosis

**DOI:** 10.1016/j.jcf.2020.09.007

**Published:** 2021-09

**Authors:** Noreen Zainal Abidin, Aaron Ions Gardner, Hannah-Louise Robinson, Iram J. Haq, Matthew F. Thomas, Malcolm Brodlie

**Affiliations:** a Translational and Clinical Research Institute, Faculty of Medical Sciences, Newcastle University, Framlington Place, Newcastle upon Tyne, NE2 4HH. United Kingdom; b Paediatric Respiratory Medicine, Great North Children's Hospital, Newcastle upon Tyne Hospitals NHS Foundation Trust, Newcastle upon Tyne, Queen Victoria Road, Newcastle upon Tyne, UK, NE1 4LP. United Kingdom

**Keywords:** Cystic fibrosis, Nontuberculous mycobacteria, Pediatrics

## Abstract

Nontuberculous mycobacteria (NTM) infection is of growing concern in cystic fibrosis (CF). UK CF Registry data were analyzed from 2016 to 2018. Prevalence of infection stabilized in the pediatric age-group during this period but remained substantially higher than in 2010. Allergic bronchopulmonary aspergillosis and *Pseudomonas aeruginosa* infection were associated with NTM infection.

## Highlights

• NTM infection in children with CF is a major clinical concern and challenge.
• Prevalence of NTM in children in the UK CF registry stabilised from 2016 to 18.
• This prevalence, however, remained substantially higher than in 2010.
• We highlight the need for high quality studies in this area.

## 1 Introduction

Respiratory infection with nontuberculous mycobacteria (NTM) has become a growing concern in children and young people with cystic fibrosis (CF) [1]. *Mycobacterium abscessus,* in particular, has been associated with an increased decline in lung function and is a contraindication to lung transplantation in many centers [2,3]. Treatment regimens for *M. abscessus* are complex, prolonged and associated with significant adverse effects. Epidemiological studies of NTM in children with CF are limited and risk factors for infection are poorly understood. Over the last 5 years there has been welcome publication of guidelines for the management of NTM-pulmonary disease [1,4]. Treatment strategies in children remain disappointingly based on extrapolated adult data, however, and there is an urgent need for high quality studies to inform more evidence-based pediatric practice.

We previously identified increasing prevalence of NTM infection in the UK pediatric CF population between 2010 and 2015, highlighting the urgent need to increase our understanding in this area [5]. Here, we expand on this work using the latest registry data, with the aim of describing recent trends in NTM infection, species-specific prevalence and identifying clinical factors associated with infection.

## 2 Methods

### 2.1 United Kingdom cystic fibrosis registry

All data were obtained following application and approval by the CF Trust Research Registry Committee. Informed and written consent is obtained from individuals or their carers for inclusion in the registry. The registry has research ethics approval and meets United Kingdom (UK) data protection regulations [6]. It captures anonymized clinical data and health outcomes on an annual basis for >90% of people with CF in the UK [7]. Here we analyzed annual review data from 4687 individuals aged ≤16 years in the UK CF Registry between 2016 and 2018.

### 2.2 Variables and data cleaning

Data fields obtained from the UK CF Registry and definitions are shown in Table 1. All annual review datasets and NTM sub-datasets were cleaned and checked for duplicates. NTM sub-datasets (containing information on species, culture dates and type of culture) were available for individuals who were recorded as having an NTM positive respiratory culture within the annual review year. These datasets were merged to enable analysis of species-specific prevalence.

**Table 1:** Variables obtained from the United Kingdom Cystic Fibrosis Registry.

Variable	Definition
Age	Age at annual review.
Gender	
Genotype	Per allele classifications of known alleles.
Postcode	Home address postcode district.
Height (cm)	As recorded at annual review date.
Weight (kg)	As recorded at annual review date.
BMI (kg/m^2^)	As recorded at annual review date.
FVC	As recorded at annual review date.
FVC% predicted	As recorded at annual review date.
FEV_1_	As recorded at annual review date.
FEV_1_% predicted	As recorded at annual review date.
Best FEV_1_	Highest value recorded within the last 12 months.
Best FEV_1_% predicted	Highest value recorded within the last 12 months.
FEF 25–75	As recorded at annual review date.
FEF 25–75% predicted	As recorded at annual review date.
Allergic bronchopulmonary aspergillosis	Patients recorded as having this diagnosis within the last 12 months.
Cystic fibrosis related diabetes	Patients recorded as having this diagnosis within the last 12 months.
*Staphylococcus aureus* status	Detected colonization at any point since the last annual review.
*Pseudomonas aeruginosa* status	Detected colonization at any point since the last annual review.
*Bukholderia cepacia*	Detected colonization at any point since the last annual review.
*Bukholderia cenocepacia*	Detected colonization at any point since the last annual review.
*Bukholderia multivorans*	Detected colonization at any point since the last annual review.
NTM status	Detected colonization at any point since the last annual review.
NTM pulmonary disease	Patients recorded as having a diagnosis of NTM pulmonary disease since the last annual review.
NTM species information	Detailed culture information for NTM culture-positive patients.
NTM treatment details	Detailed treatment information for NTM culture-positive patients.
Hospital IV antibiotics	Total number of days on IV antibiotic therapy in hospital within the last 12 months.
Home IV antibiotics	Total number of days on IV antibiotic therapy at home within the last 12 months.
Transplant evaluated	Evaluated for transplant in the last 12 months.
Transplant received	Recipient of transplant in the last 12 months.

*Abbreviations*: BMI = body mass index; FVC = forced vital capacity; FEV_1_ = forced expiratory volume in one second; FEF = forced expiratory flow; NTM = nontuberculous mycobacteria; IV = intravenous.

These procedures were performed using Microsoft Excel 2016 (Office 365, Microsoft) and R statistical software version 3.6.1 using the ‘dplyr’ package (R Foundation for Statistical Computing).

### 2.3 Statistical analysis

NTM infection was used as the dependent variable. This was determined if they had isolated NTM in a respiratory culture in the preceding year; it is not possible to ascertain form the registry data if an individual met criteria for NTM pulmonary disease. Independent variables included for analysis as ‘predictors’ of NTM infection were based on results from previous studies as well as potential confounders within limitations of data available from the UK CF Registry [1]. These were demographic data (age, gender, CF transmembrane conductance regulator genotype, body mass index) and respiratory culture results (*Staphylococcus aureus, Pseudomonas aeruginosa, Bukholderia cepacia* and *Bukholderia multivorans). S. aureus* and *P. aeruginosa* were divided into three categories: negative; intermittent, defined as 1–2 isolations in the last 12 months; chronic, defined as 3 or more isolations in the last 12 months. Lung function (forced expiratory volume in 1 second (FEV_1_) percentage predicted as calculated using the Global Lung Initiative equations) and co-morbidities (allergic bronchopulmonary aspergillosis, CF-related diabetes) were also collated.

Certain variables were excluded to avoid multi-collinearity or if numbers were too small to calculate an odds ratio as follows:

1 Height (collinear with age)
2 Weight (collinear with age)
3 *B. cepacia* (numbers too small to calculate an odds ratio)

Univariate and multivariate logistic regression analyses were performed using R statistical software, version 3.6.1. with the ‘questionr’ package (R Foundation for Statistical Computing).

Significant variables (*p*<0.05) at the univariate level were included in the initial multivariate model. We undertook a total of four elimination steps as part of a backward selection approach in developing a simplified multivariate model. The final model was chosen on the basis of the lowest Akaike Information Criterion, a bias-correcting score.

## 3 Results

**Fig. 1:**
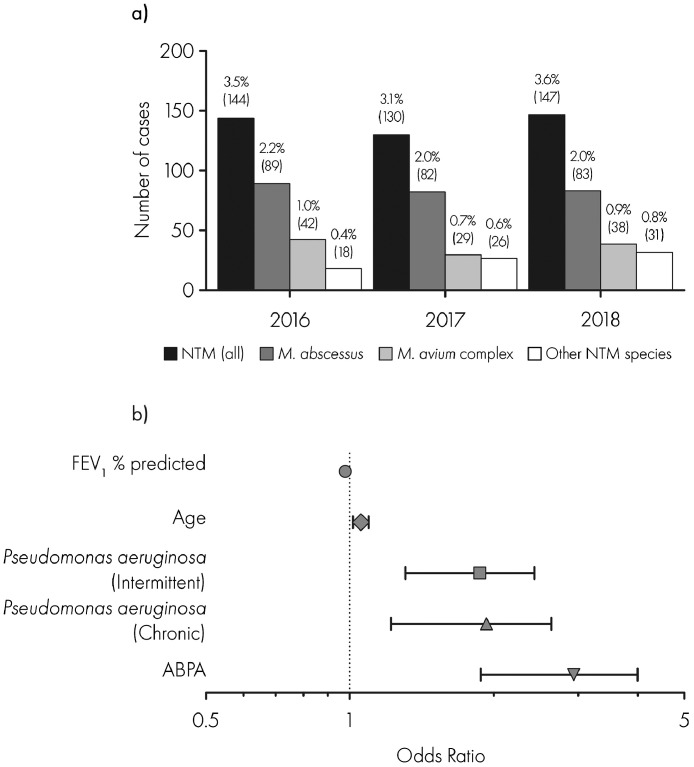
Annual prevalence of nontuberculous mycobacteria (NTM) infection in children and young people with cystic fibrosis in the United Kingdom between 2016 and 2018. NTM infection, and clinical factors significantly associated with NTM infection in the final multivariate model. a) Annual prevalence of NTM infection in children and young people with cystic fibrosis. NTM infection was defined as a case that had isolated NTM in a respiratory culture at least once in the preceding annual review year. Species-specific prevalence is shown for *Mycobacterium abscessus, Mycobacterium avium complex* and other NTM species. Percentage above each bar indicates annual prevalence with number of individual cases in brackets. b) Clinical factors significantly associated with NTM infection in the final multivariate model. Error bars represent 95% confidence intervals. Abbreviations: NTM = nontuberculous mycobacteria, FEV_1_ = forced expiratory volume in one second; ABPA = allergic bronchopulmonary aspergillosis.

Out of 4687 individuals aged less than or equal to 16 years between 2016 and 2018, 303 (6.5%) isolated NTM at least once. In terms of species, 92 (30.4%) isolated *Mycobacterium avium* complex and 176 (58.1%) *M. abscessus*. These groups were not mutually exclusive and 17 individuals isolated both species. The annual prevalence of NTM infection remained stable between 2016 and 2018 at 3.5%, 3.1% and 3.6% respectively, with similar trends in *M. abscessus* and *M. avium* complex (Fig. 1a).

In the univariate analysis, higher odds of NTM infection were associated with chronic and intermittent *P. aeruginosa* infection, allergic bronchopulmonary aspergillosis (ABPA), older age and lower FEV_1_% predicted (Table 2). CF-related diabetes and higher body mass index were also significantly associated with higher odds of NTM infection though these factors became insignificant at the multivariate level.

**Table 2:** Summary of 2016–18 merged datasets including clinical characteristics and results of univariate and multivariate logistic regression analyses.

	All			NTM			Non-NTM			Univariate		Multivariate	
	n	%	Median (IQR)	n	%	Median (IQR)	n	%	Median (IQR)	OR (95% CI)	p	OR (95% CI)	p
Patients	4687	100.0	–	303	100.0	–	4384	100.0	–				
DEMOGRAPHICS													
Age (years)	4687	100.0	9 (5–13)	303	100.0	13 (9–15)	4384	100.0	8 (4–13)	1.153 (1.123–1.186)	2.20 × 10^−16^	1.056 (1.017–1.097)	0.00474
Female	2280	48.6	–	154	50.8	–	2126	48.5	–	Reference			
Male	2407	51.4	–	149	49.2	–	2258	51.5	–	0.911 (0.72–1.15)	0.4326		
Height (cm)	4645	99.1	132.2 (108.5–155.5)	300	99.0	152 (135.2–163.2)	4344	99.1	130.8 (107.4–154.6)	Excluded			
Weight (kg)	4675	99.7	28.85 (18.36–46.3)	302	99.7	41.38 (29.24–52.27)	4373	99.7	27.8 (18–45.5)	Excluded			
Body mass index (kg/m^2^)	4644	99.1	17.07 (15.73–19.08)	302	99.7	17.75 (16.28–19.83)	4344	99.1	17.03 (15.7–19.03)	1.066 (1.028–1.104)	0.0004475		
CFTR GENOTYPE													
Other/Other	464	9.9	–	32	10.6	–	432	9.9	–	Reference			
F508del/F508del	2382	50.8	–	191	63.0	–	2191	50.0	–	1.177 (0.81–1.765)	0.41112		
F508del/Other	1841	39.3	–	80	26.4	–	1761	40.2	–	0.613 (0.405–0.948)	0.0236		
LUNG FUNCTION													
FEV_1_% predicted	3467	74.0	88.11 (75.79–98.12)	281	92.7	77.64 (65.91–89.15)	3185	72.7	88.97 (76.94–98.5)	0.971 (0.965–0.977)	2.0 × 10^−16^	0.979 (0.972–0.986)	3.83 × 10^−9^
RESPIRATORY MICROBIOLOGY													
*S. aureus* (Chronic*)	443	9.5	–	42	13.9	–	401	9.1	–	1.672 (1.623–2.353)	4.19 × 10^−3^		
*S. aureus* (Intermittent̟†)	1191	25.4	–	81	26.7	–	1110	25.3	–	1.165 (0.884–1.522)	2.70 × 10^−1^		
*P. aeruginosa* (Chronic*)	357	7.6	–	58	19.1	–	299	6.8	–	3.993 (2.872–5.487)	2.2 × 10^−16^	1.936 (1.338–2.768)	0.000361
*P. aeruginosa* (Intermittent†)	898	19.2	–	86	28.4	–	812	18.5	–	2.18 (1.653–2.858)	2.3 × 10^−8^	1.875 (1.387–2.518)	3.47 × 10^−5^
*B. cepacia*	68	1.5	–	9	3.0	–	59	1.3	–	2.244 (1.03–4.343)	2.59 × 10^−2^		
*B. cenocepacia*	11	0.2	–	0	0.0	–	11	0.3	–	Excluded			
*B. multivorans*	26	0.6	–	6	2.0	–	20	0.5	–	1.483 (0.469–3.161)	1.57 × 10^−3^		
COMORBIDITIES													
CFRD	325	6.9	–	47	15.5	–	278	6.3	–	2.712 (1.922–3.754)	4.76 × 10–^9^		
ABPA	224	4.8	–	52	17.2	–	172	3.9	–	5.073 (3.6–7.049)	2.2 × 10^−16^	2.956 (2.056–4.193)	2.32 × 10^−9^

*Abbreviations*: NTM = nontuberculous mycobacteria; IQR = interquartile range; OR = odds ratio; CI = confidence interval; FEV1 = forced expiratory volume in one second, *S. aureus* = *Staphylococcus aureus, P. aeruginosa* = *Pseudomonas aeruginiosa*, CFRD = Cystic fibrosis related diabetes, ABPA = Allergic bronchopulmonary aspergillosis. *Defined as 3 or more isolations of *Pseudomonas aeruginosa* in the last annual review year.

† Defined as 1–2 isolations of *P. aeruginosa* in the last annual review year*.*

In the final parsimonious multivariate model, age, ABPA, *P. aeruginosa* infection (chronic and intermittent) and lower FEV_1_% predicted remained significantly associated with NTM infection (Fig. 1b).

## 4 Discussion

This nationally representative study demonstrates that levels of NTM infection in children and young people with CF in the UK stabilized between 2016 and 2018. This followed a steady increase between 2010 and 2015 and levels remained substantially higher than at the start of the decade [5]. *M. abscessus* remained the predominant species. ABPA, *P. aeruginosa* infection, older age and lower lung function were associated with NTM infection.

These data are observational and can only be hypothesis-generating, however, there are several potential factors involved in this increased prevalence. Practices for screening and culture of NTM have evolved over the decade and are likely to have had some influence on identified cases. Environmental factors are important in influencing NTM prevalence but limitations of registry data did not enable investigation of this. There is also evidence of person-to-person transmission of *M. abscessus* [8]. Most recently, from 2015 to 16 infection control measures were increased and the threshold for treating *M. abscessus* infection was generally lowered in the UK [1,4].

Other limitations of our work are common to many registry studies, i.e. accuracy of data input, variation in clinical practice and reporting across centers. However, coverage is high for the UK CF Registry (>96% of CF population) and there is clear guidance to standardize data collection. Data were not available to accurately determine in individuals whether criteria for NTM pulmonary disease were present or about management strategies.

NTM infection has previously been associated with ABPA and chronic steroid therapy in CF and non-CF adult cohorts respectively [9], [10], [11], [12]. Such associations raise the interesting question of whether the immunosuppressive effect of steroids or other factors such as the impact of Aspergillus infection on the lung microbiota or associated structural lung damage predispose to NTM infection. *P. aeruginosa* was also associated with an increase in odds of NTM infection and may be a surrogate marker for increasing age and more severe lung disease. It is also possible that complex microbiological interactions between *P. aeruginosa,* Aspergillus and NTM account for some of these observations.

We conclude that the prevalence of NTM in children and young people with CF in the UK is now substantially higher than it was in 2010. Although levels appear to have plateaued latterly this remains far from reassuring and emphasizes the urgent need for well-designed trials to establish the most effective management strategies for NTM infection in children and young people with CF. These data will help inform the design of such pediatric studies.

## CRediT authorship contribution statement

**Noreen Zainal Abidin:** Formal analysis, Investigation, Methodology, Writing - original draft. **Aaron Ions Gardner:** Formal analysis, Investigation, Methodology, Writing - review & editing. **Hannah-Louise Robinson:** Formal analysis, Writing - review & editing. **Iram J. Haq:** Supervision, Writing - review & editing. **Matthew F. Thomas:** Funding acquisition, Investigation, Methodology, Supervision, Writing - review & editing. **Malcolm Brodlie:** Funding acquisition, Investigation, Methodology, Supervision, Writing - review & editing.

## Declaration of Competing Interest

None relating to this work. M.B. unrelated to this work, received investigator-led research grants from Pfizer and Roche Diagnostics; honoraria for speaking at educational meetings paid to Newcastle University from Novartis, TEVA and Roche Diagnostics; and travel and accommodation for educational meetings from Boehringer Ingelheim and Vertex Pharmaceuticals. M.F.T. unrelated to this work, received an investigator-led research grant from Pfizer. All remaining authors: No reported conflicts of interest.

## Appendix Supplementary materials

Image, application 1
