# Supplementary material for: Trends in nontuberculous mycobacteria infection in children and young people with cystic fibrosis
Source: J Cyst Fibros. 2021 Sep;20(5):737–41. doi: 10.1016/j.jcf.2020.09.007 (PMC8490157; doi:10.1016/j.jcf.2020.09.007)
Supplement: Supplementary file 1 [file mmc1.pdf]

The Newcastle upon Tyne Hospitals NHS Foundation Trust

Newcastle University

Malcolm Brodlie
MRC Clinician Scientist/Clinical Senior Lecturer
Honorary Consultant in Paediatric Respiratory Medicine

Level 3, Clinical Resource Building
Great North Children's Hospital
Royal Victoria Infirmary
Queen Victoria Road
Newcastle upon Tyne
NE1 4LP
United Kingdom
Secretary: Sandy Burlison ☎ +44 191 2820807

12 September 2020

Dear Professor Flume,

Many thanks for the opportunity to submit a revised version of our manuscript 'Trends in nontuberculous mycobacteria infection in children and young people with cystic fibrosis in the United Kingdom: a registry study'.

We are pleased that it was felt that the work will be useful in informing future paediatric studies.

Please find our response to the specific comments below:

1. Page 7, line 8: Check the reference, you state there was a steady increase in NTM infection between 2010 and 2015 but reference the management guidelines which don't contain that information. Possibly from reference 5?
   We are grateful to the reviewer for highlighting our error in referencing here, we have amended the cited reference to number 5.
2. Page 7, line 8: Could you include a reference for this sentence where prevalence is highest in 2018, do you mean for children or in all people with CF? From the previous UK registry study completed and cited (Gardner et al. 2019) the prevalence is actually higher in children in 2015 than 2018.

Thank you for pointing this out. We have now rephrased this sentence to read "This followed a steady increase between 2010 and 2015 and levels remained substantially higher than at the start of the decade.(5)"

3. Page 7, line 47-49. Could link ABPA and steroid treatment statements. Sound like two stand-alone statements rather than an association between the two.
   We have rephrased this sentences to now read:" NTM infection has previously been associated with ABPA and chronic steroid therapy in CF and non-CF adult cohorts respectively.(9-12)"

We hope that this revision is found suitable for publication.

Please do not hesitate to contact me if there are any queries or questions.

Yours sincerely

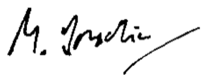

Malcolm Brodlie on behalf of all co-authors.

MRC Clinician Scientist and Clinical Senior Lecturer, Hon Consultant in Paediatric Respiratory Medicine
